# Antibody waning after immunosuppressive chemotherapy and immunomodulators, re-immunization considerations in pediatric patients with malignancy and chronic immune thrombocytopenic purpura

**DOI:** 10.1186/s12879-022-07647-1

**Published:** 2022-07-28

**Authors:** Babak Abdolkarimi, Ali Amanati, Hossein Molavi Vardanjani, Safura‏ ‏Jamshidi, Seid Amir Pasha Tabaeian

**Affiliations:** 1 Lorestan University of Medical Sciences, Khorramabad, Iran; 2 Professor Alborzi Clinical Microbiology Research Center, Shiraz University of Medical Sciences, Shiraz, Iran; 3 Research Center for Traditional Medicine and History of Medicine, Shiraz University of Medical Sciences, Shiraz, Iran; 4 Iran University of Medical Science, Tehran, Iran

**Keywords:** Catchup immunization, Hepatitis B virus, Diphtheria, Tetanus, Mumps, Measles, Rubella, Malignancy, Hematologic disorder, Immunosuppressive chemotherapy

## Abstract

**Introduction:**

Immunosuppressive chemotherapy increase the risk of vaccine-preventable infectious diseases in children; nevertheless, chemotherapy may result in delay or miss updated immunization schedules. The predictable antibody waning after incomplete primary immunization series may be intensified at the end of chemotherapy. This study aimed to investigate post-chemotherapy vaccine immunity waning at the end of immunosuppressive therapy in children with malignancy and hematologic disorders.

**Materials and methods:**

Children with malignancies and hematologic disorders including chronic immune thrombocytopenic purpura (ITP) younger than 18 years old were enrolled from September 2015 to August 2019. Eligible patients who completed their treatment protocol for at least 6 months were recruited. The patient information, including sex, age at the date of diagnosis, number of chemotherapy sessions, underlying disease, and vaccination history, was taken by chart review using predefined questionnaires. The patient’s blood samples were obtained, and serum IgG antibody titer checked against diphtheria, tetanus, hepatitis B virus (HBV), mumps, measles, and rubella (MMR) were measured by enzyme-linked immunosorbent assay (ELISA).

**Results:**

110 children receiving immunosuppressive chemotherapy were recruited. Forty-four (40%) of the children tested were girls and 66 (60%) were boys. The mean age of patients was 5.5 years with a range of 2 to 13 years. Of 110 studied children, 27.3% were seronegative for all antibodies. On average, patients undergo 19 episodes of chemotherapy. The mean chemotherapy sessions were significantly greater in children who were seronegative for all tested antibodies (mean: 36.2, 95% CI 33.16 to 39.24, p-value < 0.001). No statistically significant differences were observed regarding the patient’s sex and age between the seropositive and seronegative groups (p-value 0.513 and 0.060, respectively). Based on Poisson regression model analysis, the female gender was associated with 37% lower odds of seronegativity (incidence rate ratio (IIR): 0.63; [95% conf. interval: 0.39 to 1.01, p-value: 0.55]), while chemotherapy sessions 30 or more was associated with significant odds of seronegativity for all tested vaccines (IIR: 25.41; [95% conf. interval: 6.42 to 100.57, p-value < 0.001]).

**Conclusion:**

Our results reemphasized planned catchup immunization in children undergoing immunosuppressive chemotherapy for malignancy, especially against tetanus, diphtheria, and hepatitis B at least 6 months after the end of chemotherapy sessions.

## Introduction

Vaccination against infectious diseases is an essential part of pediatric medical care, and immunization performance in children with adequate immune function is usually guaranteed when administered according to a complete immunization schedule. Children undergoing chemotherapy for childhood cancers frequently develop acquired immunological deficiencies in cellular and humoral immunity, resulting in a reduction in vaccination protection [1, 2]. Although there is agreement on immunization for children who have had hematopoietic stem cell transplantation (HSCT), there is no universally accepted strategy for re-vaccination for children who have not had HSCT [3]. ‏The lack of re-immunization recommendations for most children receiving cytotoxic therapy but not BMT perplexes healthcare practitioners concerning the appropriate vaccine protection strategies [4]. Evidence-based recommendations should consider the degree of immunity loss during chemotherapy courses, the type of vaccine (bacterial or viral) immunity, and the optimal decision time for revaccination. Such recommendations require a thorough investigation of the various re-immunization aspects, but the available data is confusing.

It has been demonstrated that immunological improvement occurs within 6 months to 1 year after chemotherapy sessions; however, there are no universally accepted criteria for re-vaccination [5, 6].

Re-vaccination with booster dose inactivated vaccines is suggested shortly after standard-dose chemotherapy because recovered pediatric patients are susceptible to vaccine-preventable illnesses such as diphtheria and tetanus following intensive cancer treatment. In addition to inactivated vaccines, a booster dose of the attenuated live viral vaccines is recommended at least 6 months after the chemotherapy [7]. After standard-dose chemotherapy, T cell malfunction takes about a year to regenerate and normalize immunoglobulin levels [8]. T cell dysfunction can extend months, even years, after transplantation to repair cellular immunity in high-risk lymphoblastic leukemia, acute myeloid leukemia, and autologous and allogeneic bone marrow transplantation [6].

Our study aimed to examine the seroconversion rate of previously vaccinated children after the end of chemotherapy sessions in a referral oncology center in Iran.

## Material and methods

This analytical observational study was conducted between September 2015 to August 2019. Our study was conducted at the Lorestan University of Medical Science’s Pediatric Hematology/Oncology Department in Khorramabad’s Shahid Madani Hospital. All children receiving their immunosuppressive treatment according to the protocols of the pediatric oncology department who have had at least 6 months of the last chemotherapy or rituximab therapy (for chronic ITP) were included in our study. All of the included patients in the study were fully immunized according to the national primary immunization schedules.

Patients receiving a bone marrow transplant (BMT) or solid organ transplantation, children under the age of two, adults above the age of eighteen, and patients with congenital immunodeficiency were excluded. Patients having an absolute lymphocyte count (ALC) of less than 1000 per mm^3^ were also excluded from the study. Absolute lymphocyte count recovery was defined as ALC ≥ 1000/mm^3^, which patients should follow. Utilizing Sysmex KX-21 (a fully automated hematology analyzer) and a peripheral blood smear, the lymphocyte count was determined.

Each patient, or the study participants' legal guardians, completed a formal, informed consent form before inclusion in the study, as required by the Helsinki Declaration, Lorestan University of Medical Science’s Human Research Review Committee, and the Office of Human Research Support. This work was approved by Lorestan University’s Human Research Review Committee and the Human Research Protection Department (Study ID: IR.LUMS.REC.1398.170) [9].

Predefined questionnaires and direct interviews during regular visits were used to obtain clinical information on each patient, including sex, age at the time of diagnosis, age at the time of sampling, protocol risk status (standard, intermediate, or high-risk protocol), type of disease, absolute lymphocyte count at the time of the study, and vaccination history.

Serum IgG titers against diphtheria, tetanus, HBV, mumps, measles, and rubella were assessed using an ELISA kit according to the manufacturer's instructions. Around 5 mL of venous blood from each patient were collected for testing, and the sample was then analyzed in the immunological research lab using enzyme immunoassay (EIA) kits. According to the kit formula's instructions, the cut-off value was calculated using the optical density (OD) of negative control sera. Serum IgG antibody titer against measles was determined using the Enzygnost Anti-Measles Virus IgM (Dade Behring, Marburg, Germany) assay.

The test costs were paid for entirely by the project manager, with no charges placed on the patients or their families. Reactive anti-measles antibody titers were thought to be protective. Anti-mumps, anti-rubella, anti-diphtheria, and anti-tetanus titers of greater than 0.1 were deemed protective. Anti-HBV titers 10 mIU/mL or higher were found to be protective against hepatitis B. “Equivocal” results were recorded as seronegative (Table 1). Data were analyzed using SPSS statistical software version 21.0 and Stata/MP 17.0 for windows. The Mann–Whitney U test was utilized for the univariable analysis of categorical (seroprotection-status) and continuous variables (age and chemotherapy sessions) in this study because the data did not follow the normal distribution. Fisher's exact test was used to measure the comparison of proportions. An incidence risk ratio (IRR) for seronegativity was estimated based on the Poisson regression model, adjusting for gender, age groups, and chemotherapy sessions.

**Table 1:** Serological correlates of protection against tested vaccines

Vaccine type	Units	Non-reactive	Protective
Tetanus	mcg/L	< 0.1	≥ 0.1
Diphtheria	mcg/L	< 0.1	≥ 0.1
Hepatitis B	IU/mL	< 10	≥ 10
Mumps	N/A	Negative/equivocal response	Positive
Measles	N/A	Negative/equivocal response	Positive
Rubella	IU/mL	< 10	> 10

## Results

A total of one-hundred ten eligible pediatric patients were included in the study. The mean age was 5.05 ± 2.9 years (range from 2 to 13 years). There is a predominance of boys over girls (60% and 40%, respectively). The majority of studied cases have oncologic diseases, including acute lymphoblastic leukemia (ALL; 41.8%), Ewing sarcoma (3.6%), acute myeloid leukemia (AML; 1.8%), chronic myeloid leukemia (CML; 1.8%), Wilms tumor (1.8%), brain tumor (1.8%), and neuroblastoma (1.8%). 45.5% of patients had chronic ITP (50%). Before diagnosis, all patients had completed their primary immunization series, including diphtheria, tetanus, HBV, measles, mumps, and rubella. All patients evaluated at least 6 months after the end of chemotherapy.

Of 110 individuals, 30 (incidence proportion of 2.73/1000; and age-standardized incidence proportion of 2.67/1000) were fully seronegative which means that they have non-reactive results for all tested antibodies. Patients with hematologic disorders (chronic ITP) have about 62.5% lower risk of seronegativity compare with malignancy group (odds ratio: 0.375, 95% CI 0.283 to 0.498).

The incidence risk of seronegativity against tetanus, diphtheria, HBV, mumps measles, and rubella was greatest among children aged 2–5 years 35.7% (Table 2).

**Table 2:** The patient’s demographics and patient’s seroprotection status

	Seroprotection status (n, %)		Total
	Partially protected	Fully susceptible*	
Age category			
2 to 5 years	36 (64.3%)	20 (35.7%)	56
5 to 10 years	38 (82.6%)	8 (17.4%)	46
> 10 years	6 (75%)	2 (25%)	8
Total	80	30	110

^*^Represents non-reactivity of all tested vaccine-type antibodies

^**^Incidence risk

Compare with children older than 10 years and children between 2 to 5 years, children aged 5–10 years (the middle age group) have about 30% and 52% lesser risk of seronegativity (risk ratio: 0.696 and 0.478, respectively). The patient’s demographics and treatment characteristics were summarized based on the patient’s seroprotection status in Table 3.

**Table 3:** Demographic and main treatment characteristics based on patient’s seroprotection status

	Seroprotection status		p-value
	Partially protected	Fully susceptible*	
Sex			
Male (n = 66)	46 (69.7%)	20 (30.3%)	0.513^a^
Female (n = 44)	34 (77.3%)	10 (22.7%)	
Age	5.32 (SE ± 0.31)	4.33 (SE ± 0.52)	0.060^b^
Chemotherapy sessions (numbers)	13.10 (SE ± 1.18)	36.20 (SE ± 1.48)	< 0.001^b^
Malignancy versus ITP			
ITP	50 (100.0%)	0 (0.0%)	< 0.001^a^
Malignancy	30 (50.0%)	30 (50.0%)	

*Represents non-reactivity of all tested vaccine-type antibodies

^a^By Fisher's Exact Test

^b^By Mann–Whitney Test

The Poisson model was fitted based on gender, age groups, and chemotherapy sessions. Accordingly, female gender was associated with 37% lower odds of seronegativity (IIR: 0.63; [95% CI 0.39 to 1.01, p-value: 0.55]), while chemotherapy sessions 30 or more was associated with significant odds of seronegativity for all tested vaccines (IIR: 25.41; [95% conf. interval: 6.42 to 100.57, p-value < 0.001]). Considerably when the Poisson model was fitted with 10, 20, and 30 chemotherapy sessions’ cut-offs, the odds of seronegativity increased notably (IIRs 4.76, 7.15, and 25.41, respectively). Also, compared with younger children those aged over 10 years do not have an increased risk of seronegativity for tested vaccines (IIR: 1.08; [95% conf. interval: 0.80 to 1.46, p-value < 0.601]).

## Discussion

In this study, we investigated the incidence of seronegativity against diphtheria, tetanus, HBV, measles, mumps, and rubella among children with cancer and hematologic disorders mainly, chronic ITP. Non-protective antibody levels were found in more than a quarter of patients who had received immunosuppressive chemotherapy.

The impact of chemotherapy on childhood vaccine efficacy is well-known and has been thoroughly investigated. Improving immunological function after chemotherapy can take months to years for survivors of acute lymphoblastic leukemia treated with standard-dose or high-dose chemotherapy to recover the innate and adaptive immune responses. The evidence for revaccination or booster vaccinations in survivors who have not undergone HCT is of varying quality. Current evidence confirmed that seroconversion to diphtheria, tetanus, pertussis, and measles-mumps-rubella in pediatric cancer patients begins at 3 months and lasts for 12 months after the initiation of chemotherapy [10]. The loss of immunity against tetanus, measles-mumps-rubella, and HBV have been reported with varying degrees ranging from 13% to more than 50% [11].

Kovidar (1990) and Solani (2021) highlight the antibody loss following chemotherapy treatments. Others researchers are investigating other risk factors that may play a role in antibody waning following chemotherapy. Boog et al. (2020) found that younger age was associated with greater odds of antibody waning following chemotherapy. Although we were unable to establish any association between age and vaccine immunity due to the disproportional sample size in different age groups, our study has several advantages. First, the impact of chemotherapy sessions was thoroughly investigated in this study, and it was found that increasing the number of chemotherapy sessions is significantly tied to reduced vaccine immunity. Considering different chemotherapy session cut-offs and using the Poisson regression model, the odds of antibody loss increased considerably.

Moreover, we provided an updated assessment regarding common immunomodulator drugs widely used for treating chronic ITP (particularly rituximab) which indicated that, despite previous reports on mitigating *pneumococcal* and *Haemophilus influenzae* vaccine immunity after rituximab treatment, similar results could not be expected for MMR, DTP, and HBV.

Surprisingly, despite receiving a variety of immunosuppressive agent combinations such as corticosteroids and rituximab with or without cyclosporine and azathioprine, all patients with chronic ITP had protective antibody levels, whereas only 36.7% of cancer patients had protective antibody levels against all tested vaccines. As a result, while biological response modifiers (BRMs) such as rituximab (which is mainly used in patients with chronic ITP) are associated with long-term impacts on humoral immune responses, the type of underlying disease is a more crucial determinant in the waning protective antibody levels in children.

Although diminished or lost vaccination immunity has been documented during or after chemotherapy [12, 13], the long-term effects of BRMs on decreasing vaccine immunity (for both live and non-live vaccines) have received far less attention, especially in non-cancer patients [14]. In patients with hematological malignancies, the influenza vaccine response was dramatically reduced within 6 months after the last rituximab dose [15]. Rituximab treatment was reported to impair cellular and humoral immune responses to *Streptococcus pneumoniae* polysaccharide and *Haemophilus influenzae type b* (Hib) vaccines for at least 6 months [14, 16].

The tetanus toxoid immune response is mediated through a T-cell-dependent pathway, that’s not the primary mechanism of action for rituximab [17], and the usual efficacy of vaccine would be expected; however, data regarding postvaccination efficacy to tetanus toxoid is rare, and some studies indicate the opposite effect, suggesting a decreased antibody response among patients [18].

Considering data on MMR, DTP, and HBV vaccines is scarce, the results of this study can add to the body of knowledge concerning rituximab's long-term effects on vaccine immunity.

## Study limitations

The heterogeneity of immunosuppressive treatment strategies and the small number of patients are the main study limitations. Due to the small number of patients over 10 years of age as well as the possible administration of a TDP booster dose during maintenance chemotherapy in children at 4 to 6 years, observed seronegativity risks in our different age groups may not be not indicative of real protective antibody loss after completing chemotherapy courses. In addition, the patient’s antibody titers were only checked once 6 months after the end of chemotherapy; however, serial monitoring may be better for determining the best time for booster vaccine injections. We also did not have the patient’s antibody titers before starting chemotherapy, so we could not compare antibody titers before and after chemotherapy sessions. Besides, it is not possible to explore the chemotherapy agent’s effect on the vaccine immunity in this study due to the extremely high patient heterogeneity because chemotherapy regimens varied in different cancers in terms of duration of treatment, drug strength, and quantity of drugs in each protocol.

Finally, the loss of vaccine immunity can be anticipated to occur at least 6 months following the end of chemotherapy, but further research is required to find the best time for testing patient immunity and making revaccination decisions.

## Conclusion

Immunization is often overlooked in children undergoing cancer treatment. Considering sustained declines in protective antibody titers are unavoidable in patients after chemotherapy, vaccine antibody titration and re-vaccination seem necessary for pediatric oncology patients following chemotherapy. It is reasonable to begin re-vaccination of susceptible kids 6 months following the completion of chemotherapy sessions.

## Data Availability

The datasets used and analyzed during the current study are available from the corresponding author on reasonable request.

## Abbreviations

ITP: Immune thrombocytopenic purpura
HBV: Hepatitis B virus
MMR: Mumps, measles, and rubella
ELISA: Enzyme-linked immunosorbent assay
HSCT: Hematopoietic stem cell transplantation
BMT: Bone marrow transplant
ALC: Absolute lymphocyte count
AML: Acute myeloid leukemia
CML: Chronic myeloid leukemia
BRMs: Biological response modifiers
